# Hybrid Machine Learning Framework for Multistage Parkinson’s Disease Classification Using Acoustic Features of Sustained Korean Vowels

**DOI:** 10.3390/bioengineering10080984

**Published:** 2023-08-20

**Authors:** S. I. M. M. Raton Mondol, Ryul Kim, Sangmin Lee

**Affiliations:** 1 Department of Electrical and Computer Engineering, Inha University, Incheon 22212, Republic of Korea; 2 Department of Neurology, Inha University Hospital, Inha University College of Medicine, Incheon 22212, Republic of Korea

**Keywords:** voice biomarkers, multistage Parkinson’s disease, dysphonia features, machine learning classifiers

## Abstract

Recent research has achieved a great classification rate for separating healthy people from those with Parkinson’s disease (PD) using speech and the voice. However, these studies have primarily treated early and advanced stages of PD as equal entities, neglecting the distinctive speech impairments and other symptoms that vary across the different stages of the disease. To address this limitation, and improve diagnostic precision, this study assesses the selected acoustic features of dysphonia, as they relate to PD and the Hoehn and Yahr stages, by combining various preprocessing techniques and multiple classification algorithms, to create a comprehensive and robust solution for classification tasks. The dysphonia features extracted from the three sustained Korean vowels /아/(a), /이/(i), and /우/(u) exhibit diversity and strong correlations. To address this issue, the analysis of variance F-Value feature selection classifier from scikit-learn was employed, to identify the topmost relevant features. Additionally, to overcome the class imbalance problem, the synthetic minority over-sampling technique was utilized. To ensure fair comparisons, and mitigate the influence of individual classifiers, four commonly used machine learning classifiers, namely random forest (RF), support vector machine (SVM), k-nearest neighbor (kNN), and multi-layer perceptron (MLP), were employed. This approach enables a comprehensive evaluation of the feature extraction methods, and minimizes the variance in the final classification models. The proposed hybrid machine learning pipeline using the acoustic features of sustained vowels efficiently detects the early and mid-advanced stages of PD with a detection accuracy of 95.48%, and with a detection accuracy of 86.62% for the 4-stage, and a detection accuracy of 89.48% for the 3-stage classification of PD. This study successfully demonstrates the significance of utilizing the diverse acoustic features of dysphonia in the classification of PD and its stages.

## 1. Introduction

Parkinson’s disease (PD) is one of the most common neurodegenerative disorders, after Alzheimer’s disease [1]. At the initial stage of PD, symptoms typically include speech problems, tremors, and memory loss. As the disease progresses, patients may find it difficult to walk, run, communicate, and perform basic daily tasks [2]. The inability to reverse or cure the disease is its worst characteristic [3]; therefore, every effort has been made to discover it early, and take preventive steps to reduce its negative consequences. Recent studies suggest that there are currently more than 10 million people globally affected by PD [4], and it is expected to become a significant cause of mortality among the elderly population by 2040.

There is increasing evidence suggesting that individuals with PD may experience voice/speech problems during the prodromal phase [5,6,7,8,9], and such symptoms often persist into the early stages of the disease. As a result, the analysis of speech signals offers a greater possibility of detecting Parkinson’s in its early stages, and speech analysis can be used as a non-invasive and cost-effective tool in the early detection and monitoring of PD [10]. Recent studies on PD telediagnosis have focused on identifying vocal impairments through sustained vowel phonation, or running speech, in subjects [11,12,13,14,15,16,17,18,19,20,21,22,23]. These studies have employed various speech-signal-processing algorithms to extract clinically relevant data for the assessment of PD. The features derived from these data were then used to train learning algorithms, to build reliable decision support systems. Some studies have proposed the use of artificial neural networks to distinguish healthy individuals from those with PD, while others have suggested the use of simple speech-signal-processing algorithms.

For example, Sakar et al. [11] were the first to introduce the tunable Q-factor wavelet transform (TQWT) to extract features from the voice signals of PD patients. Using ensemble learning approaches that combine different machine learning classifiers, their results demonstrate that TQWT performs comparably better than state-of-the-art speech-signal-processing algorithms in PD classification. Avuçlu et al. [12] employed multiple classifiers to detect PD, using 22 vocal features from 195 sound samples in various training and testing instances. They utilized signal-processing techniques to extract important features from the acoustic signals of subjects with Parkinson’s, and control subjects. Bourouhou et al. [13] also evaluated a variety of classifiers to identify people who might have PD. Similarly, Zhang et al. [14] used naive Bayes, as well as other machine learning methods, to identify the presence of PD. Meghraoui et al. [15] suggested using Bernoulli and Multinomial Naive Bayes (BMNB) with harmonicity, pitch, and pulse features to identify PD. Braga et al. [16] proposed a methodology to detect early signs of PD through free speech in uncontrolled background conditions, using signal- and speech-processing techniques integrated with machine learning algorithms with a very high accuracy. Nilashi et al. [17] employed a new hybrid intelligent system for predicting PD progression, using noise removal, clustering, and prediction methods. They used the adaptive neuro-fuzzy inference system and support vector regression to predict PD progression. Dao et al. [18] proposed an approach that uses grey wolf optimization for feature selection, and the light gradient-boosting machine for classification. Kadiri et al. [19] presented a technique for identifying PD by utilizing SVM with single-frequency filtering cepstral coefficients and shifted delta cepstral features derived from the voice signals of both Parkinson’s patients, and control subjects. Jie et al. [20] employed a deep learning algorithm to learn from PD speech data. They used an embedded deep stack group sparse autoencoder for deep feature learning, and the resulting features were combined with the original speech features. Gunduz et al. [21] proposed two frameworks, based on convolutional neural networks, to classify PD using vocal features. The frameworks differ in how they combine feature sets. Deep features successfully distinguish PD patients from healthy individuals, and improve the classifier performance. While the results may appear impressive, their approach does not seem practical for several reasons. All these previous works focused on binary classification, treating the early and advanced stages of PD equally. However, the multistage classification of PD to assess new characteristics for neurocognitive assessment is important. It involves dividing patients into different stages, based on symptom severity, and developing separate classification models for each stage. Considering the different stages of PD and their associated symptoms, this approach could enable better treatment outcomes.

Only a few studies have investigated the use of a multistage classification in PD. For example, Hsu et al. [24] utilized photon emission computed tomography, and Ricciardi et al. [25] employed a three-dimensional gait analysis in their research into the multistage classification of PD. However, it is important to uncover whether voice/speech acoustic characteristics can be useful in a multistage classification. Suppa et al. [26] evaluated voice changes using machine learning algorithms in patients with PD at different stages of the disease, while receiving OFF and ON therapy. However, this research aimed to investigate the impact of the disease severity on the voice, to explore the effect of L-Dopa medication on groups of patients at the early stage (H&Y ≤ 2), and the mid–advanced stage (H&Y > 2). Similarly, Templeton et al. [27] conducted a study utilizing various neurocognitive functional tests, including speech, to classify individuals into the early stages (H&Y Stages 1 and 2) and advanced stages (H&Y Stages 3, 4, and 5) of PD. However, none of the approaches focused on a multistage classification. Instead, they were limited to a binary classification, specifically classifying between two groups of disease severity.

Therefore, the objective of this study is to use a hybrid machine learning pipeline (HMLP) to evaluate new characteristics obtained from neurocognitive assessments, also known as digital biomarkers, in relation to PD and its stages (H&Y Stages 1–5) [22]. The proposed HMLP was used in stage classification, to effectively classify the stages of PD as they are in.

The main contributions of this article are as follows:

(i) A speech corpus of three sustained Korean vowels has been created (InhaPD) from Parkinson’s patients who were recruited at Inha University Hospital.
(ii) A feature bank consisting of 43 acoustic features has been collaboratively created, using baseline features, vocal-fold features, and time–frequency features. The analysis of variance (ANOVA) F-Value feature selection classifier from scikit-learn was employed, to identify the top 20 most relevant features, resulting in a more effective and efficient feature subset.
(iii) To address the dataset imbalance, we introduced a synthetic minority over-sampling technique (SMOTE) that generates new samples in the minority class and its neighboring samples, effectively balancing the distribution of classes, and reducing bias in the classification.
(iv) A 10-fold cross-validation is performed for each model, using StratifiedKFold, demonstrating the robustness and effectiveness of our system compared to other recent approaches to stage classification.
(v) The evaluation of the classification included the measuring accuracy, precision, recall, F1 score, and the area under the curve (AUC). This evaluation aimed to test the hypothesis proposing that a HMLP analysis of sustained vowels could effectively differentiate individuals with different stages of the disease.

The remainder of this paper is structured as follows. Section 2 explains our methodology, and the architecture of the proposed framework. Section 3 presents the experimental results. Section 4 discusses the findings and limitations of the research, and Section 5 concludes the paper.

## 2. Methodology

Currently, there is a lack of a standardized methodology that has undergone sufficient validation through clinical trials [23]. The primary focus of this research was not to develop a machine learning algorithm solely aimed at achieving the highest accuracy in automatically detecting PD. Instead, the study aimed to isolate multiple factors, and carry out experiments to determine whether these factors might lead to overly optimistic outcomes in the multistage classification of PD. The details of each part can be found in the subsequent sections.

### 2.1. Methods

**Figure 1:**
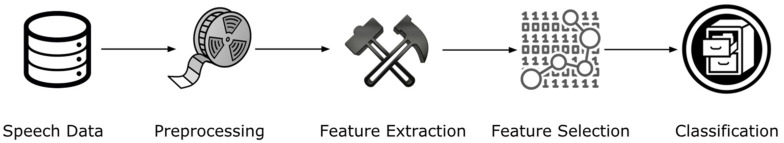
Overview of the proposed PD classification system.

This study examines speech signals, and extracts relevant features to correlate with responses, including several classes of PD. The disease detection model, depicted in Figure 1, involves multiple steps, from voice recording to the final classification. The primary steps include data collection, feature extraction and selection, model training for classification, and validation. The performance metrics of the classifier are evaluated, to determine the success of the detection system.

### 2.2. Dataset

In the areas of expanding datasets to include a larger corpus, and discovering novel objective biomarkers [28], a new corpus has been created, named InhaPD. The voice data for InhaPD were obtained from Parkinson’s patients who were recruited at Inha University Hospital, Incheon, South Korea. We enrolled a total of 101 individuals diagnosed with PD, who were between the ages of 43 and 81 years, with an average age of 68.11 ± 6.83 years. In all cases, the participants were native Korean speakers. None of the participants reported any respiratory disorders, hearing loss, or non-neurological disorders that may affect their vocal cords. All participants provided written informed consent, which was authorized by the institutional ethics committee. The clinical diagnosis of PD was based on the UK PD Society Brain Bank Diagnostic Criteria [29]. The symptoms and signs linked to Parkinson’s disease were evaluated and scored utilizing the H&Y scale [22]. Table 1 gives a comprehensive overview of the demographic details of the PD patients, including their gender, age, age onset, disease duration, and H&Y stage. The full data set can be found in the Supplementary Materials.

### 2.3. Voice Recordings

Consumer-focused devices, such as smartphones or tablets, offer an alternative that is widely available to patients, and has the potential to provide objective, frequent, and sensitive assessments. Additionally, the remote monitoring of PD using smart devices is gaining popularity, and numerous recent studies have explored the use of mobile devices for voice recording in PD research. For instance, Omberg et al. [30] used an iPhone for their voice recordings, while Farago et al. [31] employed Android smartphone devices (model not specified). On the other hand, Asci et al. [32] utilized various smartphones available on the market (Apple^®^, Samsung^®^, Huawei^®^, Xiaomi^®^, and Asus^®^). Lipsmeier et al. [33] used a Samsung Galaxy S3 mini; similarly, Vaiciukynas et al. [34] used a Samsung Galaxy Note 3, and Benba et al. [35] used a sound card in a desktop computer. These studies suggest that mobile devices can be a feasible and reliable alternative to traditional recording equipment for voice recording in Parkinson’s disease research.

In this study, the recordings were conducted in the inspection room of the hospital, using a Samsung Galaxy Tab S7 FE placed approximately 30 cm away from the mouth. The recording was not performed in an anechoic chamber, so a low level of ambient noise was present. The noise, however, was not significant enough to interfere with the analysis of the voice recordings. Similar to the approach followed by Omberg et al. [30] and Asci et al. [32], we also saved our voice samples in a compressed audio file format. The voice samples were recorded in mp3 format, with a sampling rate of 48 kHz, and a 32-bit sample size.

To obtain the voice recordings, participants were instructed to perform specific speech tasks using their normal voice intensity, pitch, and quality. The speech tasks included the sustained production of three Korean vowels, namely, /아/(a), /이/(i), and /우/(u). Each subject was recorded producing these vowels for a minimum of 10 s, resulting in a total of nine voice recordings for each subject (three samples for each vowel). The recorded Korean vowels were equivalent to the English /a/, /i/, and /u/, correspondingly.

### 2.4. Acoustic Measures

The research focusing on the impact of PD on the phonatory system mainly investigates the dysfunctions observed in the structures and muscles responsible for phonation. These include the diaphragm, muscles associated with the larynx, the vocal folds, and the resonant cavities above the glottis. Utilizing sustained vowels can produce simple acoustic patterns that have the potential to yield consistent and dependable assessments of voice quality [28]. These acoustic measures can be directly acquired using software and libraries that are readily accessible in the literature [30,36]. In this study, a set of 43 widely known acoustic measures were extracted for each vowel. All these features and their definitions are listed in Table 2.

Among these measures, all F0 features, all F1–4 features, all the jitter features, all the shimmer features, HNR, pF, F_Disp, avgF, Fitch_vtl, delta_F, and vtl_delta_F were obtained from Parselmouth 0.3.3 [37], which is a Python library for Praat software [36]. The mel-frequency cepstrum related features, for example, MFCC 1–4, and MFCC jitter 1–4, were obtained using MATLAB, as mentioned in [30].

### 2.5. Feature Selection

**Figure 2:**
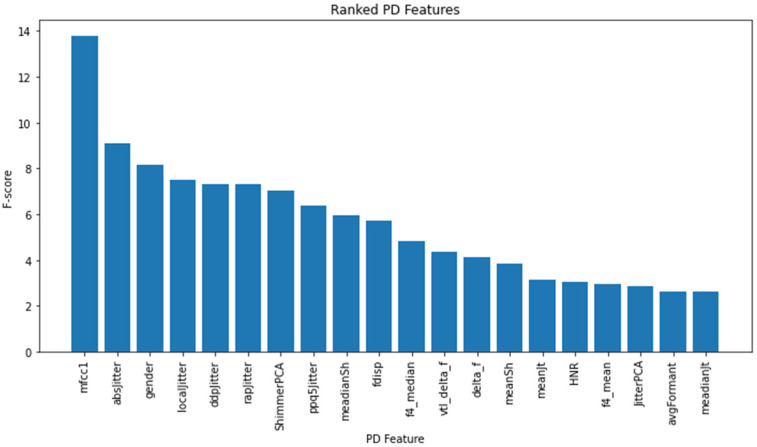
Feature ranking based on F-test scores.

Most experiments, especially those involving larger corpora where the dimensionality of the feature vectors exceeded the number of recordings, utilized dimensionality-reduction techniques. For instance, Ozbolt et al. [23] used principal component analysis for dimensionality reduction in their study, utilizing the scikit-learn Python module. They also employed the ANOVA F-Value feature selection classifier from the scikit-learn SelectKBest module [38], to identify the most informative features for PD detection, selecting 10, 30, 50, or 70 features. In our case, k is set to 20, meaning that the top 20 features with the highest F-test scores were selected. Considering a total of 43 extracted acoustic features, along with the inclusion of gender information as an additional feature, the study utilized a set of 44 features for evaluation. Notably, no dimensionality-reduction techniques were applied in this regard. The ranking of the 20 selected features for the multistage (stage 1 to 3) classification is depicted in Figure 2. It should be noted that the feature ranking may vary for different classifications, as different stages of PD might have varying acoustic characteristics. However, it is important to emphasize that feature ranking is not the primary focus of this research; rather, it is utilized for analysis and classification purposes.

### 2.6. Data Balancing

Class imbalance is a common concern in clinical data, where there is a substantial difference in the number of patients and control group samples. This imbalance can create challenges in a machine learning classification, leading to a biased accuracy that favors the majority class. To address this issue, Chawla et al. [39] introduced the synthetic minority over-sampling technique (SMOTE), which generates new samples in the minority class and its neighboring samples, effectively balancing the class distribution, and mitigating classification bias. In this study, we also employed SMOTE to address our class-imbalance problem concerning the different stages of PD.

### 2.7. Machine Learning Algorithms and Hyperparameters

After selecting the top-ranked features, and addressing the issue of data imbalance, this study employed four commonly used machine learning classifiers from the scikit-learn Python module [38]: RF, SVM, kNN, and MLP. These groups of algorithms are common, due to their high model interpretability, effective minimization of misclassification, and strong diagnostic performance. While many studies do use DNN, simpler models are often preferred, due to the limited size of the available datasets [23].

Random forest (RF) is an ensemble learning algorithm that combines multiple decision trees to improve the performance and reduce overfitting. It works by randomly selecting a subset of features and samples to build each tree, and then aggregating the predictions of all the trees to make the final decision.

Support vector machine (SVM) is a supervised learning algorithm that can be used in both regression and classification tasks. It is particularly useful for datasets with complex features, and can handle both linear and nonlinear data. SVM works by finding the best separating hyperplane that maximizes the margin between the different classes.

K-nearest neighbor (kNN) is a simple and intuitive algorithm that can be used both in classification and in regression tasks. It works by finding the k-closest neighbors of a new sample, and then assigning it to the most common class among those neighbors. The value of k is usually chosen through cross-validation.

Multi-layer perceptron (MLP) is a type of artificial neural network that consists of multiple layers of interconnected nodes, also known as neurons. Each neuron applies a nonlinear activation function to the weighted sum of its inputs, allowing the network to learn complex relationships between the input features and the target variable.

A 10-fold cross-validation is performed for each model, using StratifiedKFold from the scikit-learn Python module. In 10-fold cross-validation, the data are divided into ten subsets, and each subset is used as a validation set exactly once, while the remaining nine subsets are used as the training set. This process is repeated ten times, with each of the ten subsets used exactly once, as the validation data. GridSearchCV is also used to find the best hyperparameters for each model, through searching the specified hyperparameter grid, and evaluating the model performance using cross-validation. The performance metrics are calculated for each fold, and the average performance metrics are reported for each model.

### 2.8. Performance Metrics

Choosing the appropriate metrics is crucial, because they affect the way in which performance is measured and compared. For instance, accuracy can be a useful metric when the classes are balanced but, when the classes are imbalanced, it can be misleading. In such cases, metrics such as precision, recall, F1 score, confusion matrix, and AUC can provide a better understanding of the model’s performance [40].

Accuracy measures the overall correctness of the model’s predictions. Precision (also known as the positive predictive value (PPV)) quantifies the proportion of correctly classified positive samples (PD stages) out of the total predicted positive samples, while recall (also known as sensitivity) measures the proportion of correctly classified positive samples out of the total actual positive samples. The F1 score provides a balanced measure, by taking into account both precision and recall.

To summarize the model’s performance, a confusion matrix is commonly utilized. It consists of four components: true positives (TP), true negatives (TN), false positives (FP), and false negatives (FN). True positives represent cases where the model correctly predicts the positive class, or the correct stage of PD. True negatives refer to cases where the model correctly predicts the negative class, or correctly identifies a different PD stage. False positives occur when the model incorrectly predicts a positive class, or wrongly identifies a PD stage, while false negatives refer to cases where the model incorrectly predicts a negative class, or fails to identify a PD stage.

These performance metrics, including accuracy, precision, recall, and the F1 score, along with the confusion matrix, provide valuable insights into the algorithm’s effectiveness in classifying different stages of Parkinson’s disease. Additionally, AUC is a commonly used metric that quantifies the classifier’s discrimination ability in ranking and assigning higher probabilities to positive instances, compared to negative instances.

## 3. Results

This section presents the results obtained using the Parkinson’s disease (PD) datasets defined in Section 2.2. The machine learning classifiers used in this study included random forest (RF), support vector machine (SVM), k-nearest neighbor (kNN), and multi-layer perceptron (MLP). After preprocessing the dataset, which involved feature selection and data balancing techniques, we obtained the best hyperparameters for each model, and evaluated their performance. The best hyperparameters for each algorithm have also been listed in the tables. Five evaluation metrics were used: accuracy, precision, recall, F1 score, and AUC. We evaluated the results in two subsequent sections, namely Section 3.1 and Section 3.2, using the binary and multistage approaches, respectively.

### 3.1. Binary Classification

**Figure 3:**
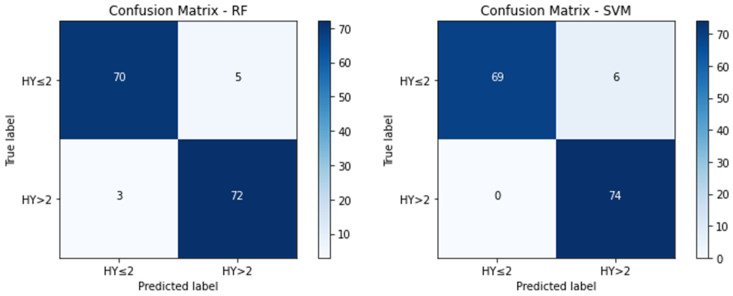

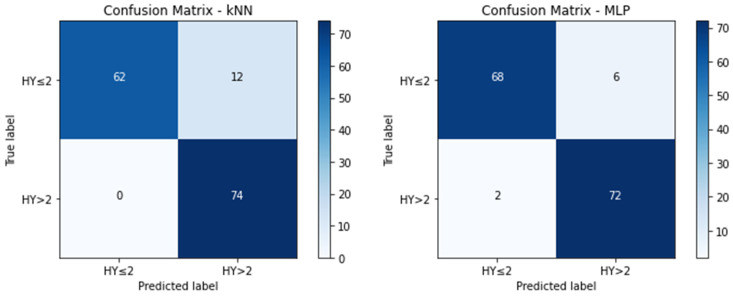
Confusion matrix for the proposed HMLP models for HY ≤ 2 vs. HY > 2 classification.

The classification of disease stages, specifically distinguishing between early-stage (H&Y Stages 1 and 2) and mid-stage (H&Y Stage 3) Parkinson’s disease, was conducted for all individuals with PD. Table 3 reports the measured metrics for the classification between individuals in the early stage and mid-stages of PD. SVM demonstrates the highest accuracy of 95.48%, and an F1 score of 0.9528, kNN produces the highest precision of 0.9956, and RF yields the highest recall of 0.9322, and an AUC score of 0.9873. However, MLP’s performance is relatively weaker, compared to the other models in the binary classification. Figure 3 resembles their corresponding confusion matrix.

Figure 3 represents the confusion matrix for the proposed HMLP models for HY ≤ 2 vs. HY > 2 classification. The confusion matrix also supports the analysis in Table 3.

Suppa et al. [26] and Templeton et al. [27] conducted an analysis comparing individuals with HY ≤ 2 (H&Y stages 1 and 2) to those with HY > 2 (H&Y stage 3 and/or more) in their respective research studies. Table 4 presents a comparative analysis of the results, highlighting the superior performance of our proposed HMLP approach utilizing the SVM algorithm. The results indicate that our approach achieved a higher accuracy, precision, and AUC, compared to the referenced studies. Notably, none of the studies were able to attain an accuracy level of 90% or higher.

### 3.2. Multistage Classification

**Figure 4:**
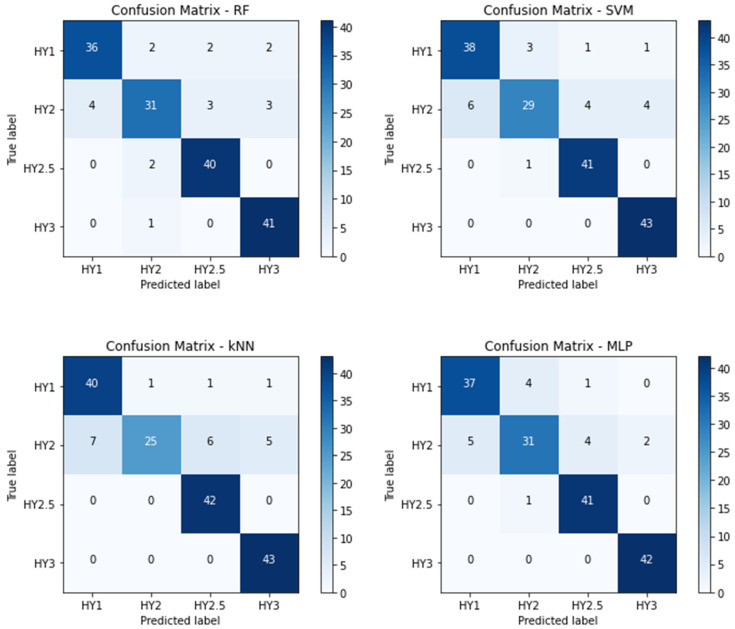
Confusion matrix for 4-stage (stage 1, 2, 2.5, and 3) classification.

**Figure 5:**
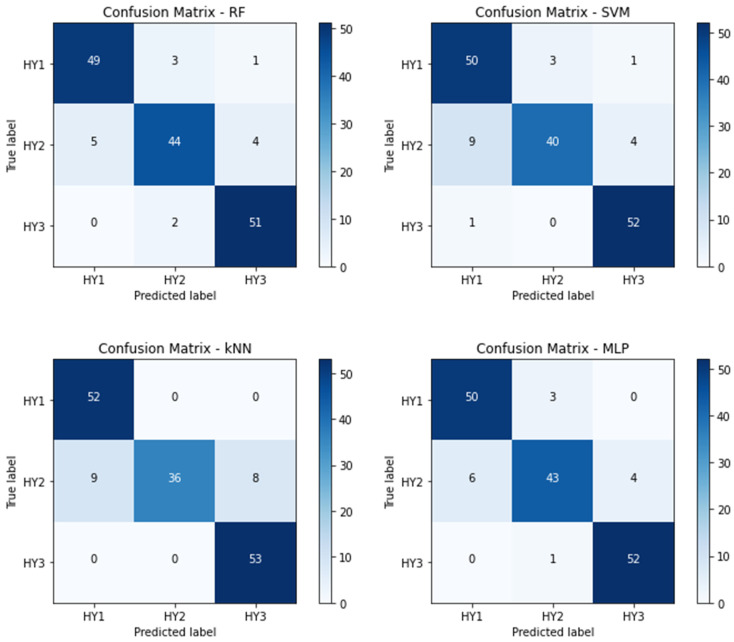
Confusion matrix for 3-stage (stage 1, 2, and 3) classification.

The analysis mentioned above falls into the category of a binary classification. However, the primary objective of this study was to perform a multistage classification of PD. As a result, the classification of the disease stages (i.e., determining the stage of PD) was conducted for all individuals with Parkinson’s. The performance metrics in Table 5 correspond with the 4-stage classification, whereas Table 6 corresponds to the 3-stage classification. Figure 4 and Figure 5 represent their corresponding confusion matrices, respectively.

Figure 4 represents the confusion matrix for the proposed HMLP models for 4-stage (stage 1, 2, 2.5, and 3) classification. The confusion matrix also supports the analysis in Table 5.

Figure 5 represents the confusion matrix for the proposed HMLP models for a 3-stage (stage 1, 2, and 3) classification. The confusion matrix also supports the analysis in Table 6.

In all scenarios, the accuracy of the multistage classification was consistently lower compared to binary classification, with accuracy levels reaching approximately 90%. Nevertheless, it is important to highlight that MLP, despite exhibiting a lower performance in the binary classification, surpassed all the other algorithms in the multistage classification tasks.

## 4. Discussion

The primary objective of this research was not to develop a machine learning algorithm solely focused on achieving the highest accuracy in the multistage classification of Parkinson’s disease (PD). Instead, the study aimed to isolate multiple factors, and conduct experiments to determine whether these factors might lead to overly optimistic results in the multistage classification of PD. Using voice biomarkers in machine learning methods, we aim to explore a non-invasive and cost-effective diagnostic tool for better treatment outcomes, to improve the quality of the patient’s life.

Furthermore, machine-learning-based voice analysis may also hold potential for assessing the effectiveness of symptomatic treatments for PD. By comparing the pre- and post-treatment voice characteristics, machine learning algorithms can potentially determine whether a treatment has improved the patient’s motor function and overall quality of life. Recent advancements in speech analysis methodologies have shown promising results. We proposed a hybrid machine learning pipeline consisting of four commonly used ML classifiers, using the acoustic features of sustained Korean vowels, and found that the hybrid model outperforms the baseline models. We achieved an accuracy of 86.62%, 89.48%, and 95.48% for the 4-stage, 3-stage, and 2-stage classification of PD, respectively, which was higher than state-of-the-art methods. However, the multistage accuracy of our model may be limited by the size of the dataset used in the evaluation. Using a larger dataset could improve the model’s ability to generalize and classify the stages of PD more effectively. Previous studies have investigated the multistage classification of Parkinson’s disease using invasive methods, such as photon emission computed tomography [24] and three-dimensional gait analysis [25]. However, it is crucial to determine the potential of non-invasive methods, specifically voice/speech acoustic characteristics, in a multistage classification. Suppa et al. [26] examined voice changes in patients with Parkinson’s disease using machine learning algorithms, specifically investigating the impact of the disease severity on the voice and the effect of L-Dopa medication on patients at the early stage (H&Y ≤ 2) and the mid–advanced stage (H&Y > 2). Similarly, Templeton et al. [27] conducted a study utilizing neurocognitive functional tests, including speech, to classify individuals into the early stages (H&Y Stages 1 and 2) and the advanced stages (H&Y Stages 3, 4, and 5) of PD. However, both studies were focused on a binary classification, and did not address a multistage classification that distinguished between multiple levels of disease severity. In summary, our study presents a reliable model for detecting PD and its stages with higher accuracy, precision, recall, F1 score, and AUC rates.

Overall, the usefulness of machine-learning-based voice analysis in PD should be further explored and discussed. It should be noted that there are several limitations to the present study. Firstly, as we did not record vocal samples from each patient sequentially, it is possible that there may be daily fluctuations in the vocal characteristics in PD that we were unable to capture. Moreover, in terms of clinical–instrumental correlations, it is important to acknowledge that machine learning analysis requires a large number of data. Furthermore, the relationship between specific aspects of hypokinetic dysarthria in PD (such as hypophonia, mono-pitch, and mono-loudness speech), and the specific voice features selected by the machine learning algorithm, remains uncertain, and requires further investigation. While k-fold cross-validation offers advantages, such as optimizing the use of limited data, and ensuring model robustness across multiple subsets, there is the inherent risk of overfitting when the entire dataset is used. It is vital to understand that, while cross-validation might produce optimistic results, these may not always mirror the real-world performance in a new, independent cohort. Relying exclusively on cross-validation results could lead to inflated expectations. An alternative, more conservative method involves partitioning the dataset into distinct training and testing sets. This holdout method minimizes the overfitting impact by evaluating the model’s performance on an untouched test set. In contrast, using a holdout dataset offers a more genuine glimpse into how the model is likely to fare when presented with new, real-world data, despite our data limitations necessitating the use of cross-validation. To develop a dependable dataset for detecting PD using acoustic features, it is essential to consider some other factors, including the gender and age balance, the quality of the microphone, noise, robustness, the number of subjects, the disease severity, and the impact of PD medication.

The current understanding of speech disorders in PD is primarily based on perceptual speech assessment. The computerized analysis of speech or the voice has been suggested for the diagnosis and monitoring of PD. Despite the potential advantages of voice analysis in PD, it has not been widely adopted in standard clinical practice yet [10]. Besides, machine learning models for detecting Parkinson’s disease are usually evaluated based on standard machine learning parameters, such as accuracy, precision, recall, F1 score, and AUC rates. However, these parameters may not accurately reflect the clinical effectiveness and anticipated positive adjustments to patient treatment. Additionally, doctors require training on how to utilize AI-powered diagnostic tools, as some of these parameters can be complex to interpret.

## 5. Conclusions

We anticipate that machine-learning-based voice analysis could serve as a novel disease biomarker in the near future, facilitating the clinical management of PD. Furthermore, we believe that our study could encourage the future use of machine learning voice analysis in PD telemedicine approaches, including home-based applications. We also speculate that future studies with a more sensible dataset may report a higher multistage accuracy than that reported in this study.

## Figures and Tables

**Table 1:** Demographic information of InhaPD.

	PD Stages				
	Whole Group	H&Y 1	H&Y 2	H&Y 2.5	H&Y 3
Number	101	23	49	12	17
Gender					
Male	60	13	28	11	8
Female	41	10	21	1	9
Age					
avg	68.11	67.08	67.57	71.08	69
std	6.83	7	7.15	3.89	7.1
min	43	49	43	65	54
max	81	81	80	80	79
Age onset					
avg	62.86	65.47	62.59	65.41	58.29
std	7.79	7.09	7.75	5.23	8.61
min	41	47	41	59	43
max	78	78	76	75	71
Disease duration					
avg	5.33	1.86	5	5.75	10.7
std	4.14	1.01	3.29	2.98	4.35
min	1	1	1	1	1
max	18	4	15	11	18

NB. avg (average), std (standard deviation), min (minimum), max (maximum).

**Table 2:** A summary of the feature sets used in this study.

Feature Name	Definitions	Symbol
F0 (mean)	Mean of the fundamental frequency	meanF0
F0 (median)	Median of the fundamental frequency	medianF0
F0 (stdv.)	Standard deviation of the fundamental frequency	stdevF0
F 1–4 (mean)	Mean of the formant frequencies F1 to F4	f1-4_mean
F 1–4 (median)	Median of the formant frequencies F1 to F4	f1-4_median
Jitter (%)	Measure of the average absolute difference between consecutive periods, divided by the average period	localJitter
Jitter (abs)	Measure of the cycle-to-cycle variation in the fundamental frequency, typically expressed in seconds	absJitter
Jitter: RAP	Measure of the average absolute difference between a period and the average of that period and its two neighboring periods, divided by the average period length	rapJitter
Jitter: PPQ5	Measure of the average absolute difference between a period and the average of that period and its four closest neighboring periods, divided by the average period length	ppq5Jitter
Jitter: DDP	Measure of the average absolute difference between consecutive differences of consecutive periods, divided by the average period length	ddpJitter
Jitter: mean	Mean of the jitter	meanJt
Jitter: median	Median of the jitter	medianJt
Jitter: PCA	Two-factor principal components analysis (PCA) on jitter	jitterPCA
Shimmer (%)	Measure of the average absolute difference between the amplitudes of consecutive periods, divided by the average amplitude, expressed as a percentage	localShimmer
Shimmer (dB)	Measure of the average absolute base-10 logarithm of the difference between the amplitudes of consecutive periods, multiplied by 20	dbShimmer
Shimmer: APQ3	Measure of the variability in the amplitude of a speech signal, as measured from the amplitude of a single period to the average amplitude of its three closest neighbors	apq3Sh
Shimmer: APQ5	Measure of the variability in the amplitude of a speech signal, as measured from the amplitude of a single period to the average amplitude of its five closest neighbors	apq5Sh
Shimmer: APQ11	Measure of the variability in the amplitude of a speech signal, as measured from the amplitude of a single period to the average amplitude of its eleven closest neighbors	apq11Sh
Shimmer: DDA	Measure of the variability in the amplitude of a speech signal, as measured from the consecutive differences between the amplitudes of consecutive periods	ddaSh
Shimmer: mean	Mean of the shimmer	meanSh
Shimmer: median	Median of the shimmer	medianSh
Shimmer: PCA	Two-factor PCA on shimmer	shimmerPCA
HNR	Amplitude of the tonal relative to the noise components.	hnr
MFCC 1–4	Mel-frequency cepstrum band 1 to 4	mfcc1-4
MFCC jitter 1–4	Positive change in mel-frequency cepstrum band 1 to 4 over time	mfccJt1-4
pF	Formant position	pF
F_Disp	Dispersion of formant frequency	fdisp
avgF	Average formant	avgFormant
Fitch_vlt	Fitch vocal tract length	fitch_vtl
delta_F	Cumulated variation of formants	delta_f
vtl_delta_F	Cumulated variation of vocal tract length	vtl_delta_f
Gender	Gender information of each subject (male, female)	gender

**Table 3:** Performance on the proposed HMLP models for HY ≤ 2 vs. HY > 2 classification.

Model	Best Hyperparameters	Average Accuracy	Average Precision	Average Recall	Average F1 Score	Average AUC
RF	{‘max_depth’: None, ‘min_samples_split’: 2, ‘n_estimators’: 200}	0.9448	0.9575	0.9322	0.9438	0.9873
SVM	{‘C’: 50, ‘gamma’: ‘scale’}	0.9548	0.9914	0.9176	0.9528	0.9846
kNN	{‘n_neighbors’: 3, ‘weights’: ‘distance’}	0.9162	0.9956	0.8364	0.908	0.9694
MLP	{‘activation’: ‘relu’, ‘hidden_layer_sizes’: (1000, 500, 200), ‘learning_rate’: ‘constant’, ‘max_iter’: 3000, ‘solver’: ‘adam’}	0.9369	0.963	0.9097	0.9348	0.9785

**Table 4:** Comparison of the results for HY ≤ 2 vs. HY > 2 classification.

Comparisons	Accuracy	Precision (PPV)	Recall (Sensitivity)	AUC	Algorithm	Authors
HY ≤ 2 vs. HY > 2	0.9548	0.9914	0.9176	0.9848	SVM	Proposed HMLP
	0.880	0.889	0.872	0.934	SVM and ANN	Suppa et al. [26]
	0.8947	0.9286	0.9286	-	CART	Templeton et al. [27]

N.B.: HY ≤ 2 (early-stage patients with PD, H&Y Stage ≤ 2), HY > 2 (mid–advanced-stage patients with PD, H&Y Stage > 2), SVM (support vector machine), ANN (artificial neural network), CART (classification and regression tree), (-) not available.

**Table 5:** Performance on the proposed HMLP models for 4-stage (stage 1, 2, 2.5, and 3) classification.

Model	Best Hyperparameters	Average Accuracy	Average Precision	Average Recall	Average F1 Score	Average AUC
RF	{‘max_depth’: None, ‘min_samples_split’: 2, ‘n_estimators’: 200}	0.8503	0.8514	0.8503	0.8474	0.9694
SVM	{‘C’: 50, ‘gamma’: ‘scale’}	0.8651	0.8671	0.8651	0.8604	0.9625
kNN	{‘n_neighbors’: 3, ‘weights’: ‘distance’}	0.8611	0.8696	0.8611	0.8513	0.9469
MLP	{‘activation’: ‘relu’, ‘hidden_layer_sizes’: (1000, 500, 200), ‘learning_rate’: ‘constant’, ‘max_iter’: 3000, ‘solver’: ‘adam’}	0.8662	0.8662	0.8662	0.8635	0.9584

**Table 6:** Performance on the proposed HMLP models for 3-stage (stage 1, 2, and 3) classification.

Model	Best Hyperparameters	Average Accuracy	Average Precision	Average Recall	Average F1 Score	Average AUC
RF	{‘max_depth’: None, ‘min_samples_split’: 2, ‘n_estimators’: 200}	0.8868	0.8887	0.8868	0.886	0.9764
SVM	{‘C’: 50, ‘gamma’: ‘scale’}	0.8764	0.8819	0.8764	0.8739	0.9603
kNN	{‘n_neighbors’: 3, ‘weights’: ‘distance’}	0.8752	0.8898	0.8752	0.8695	0.9533
MLP	{‘activation’: ‘relu’, ‘hidden_layer_sizes’: (1000, 500, 200), ‘learning_rate’: ‘constant’, ‘max_iter’: 3000, ‘solver’: ‘adam’}	0.8948	0.8964	0.8948	0.8933	0.9602

## Data Availability

The simulation experiment data used to support the findings of this study are available as supplementary material, attached to the manuscript.

## Supplementary Materials

The following supporting information can be downloaded at: https://www.mdpi.com/article/10.3390/bioengineering10080984/s1, A “Dataset Description.txt” file has also been included mentioning all the details about the included files.
